# Proximity to criticality predicts surface properties of biomolecular condensates

**DOI:** 10.1073/pnas.2220014120

**Published:** 2023-05-30

**Authors:** Andrew G. T. Pyo, Yaojun Zhang, Ned S. Wingreen

**Affiliations:** ^a^Department of Physics, Princeton University, Princeton, NJ 08544; ^b^Department of Physics and Astronomy, Johns Hopkins University, Baltimore, MD 21218; ^c^Department of Biophysics, Johns Hopkins University, Baltimore, MD 21218; ^d^Lewis-Sigler Institute for Integrative Genomics, Princeton University, Princeton, NJ 08544; ^e^Department of Molecular Biology, Princeton University, Princeton, NJ 08544

**Keywords:** biomolecular condensates, phase separation, critical phenomena, surface tension, molecular-dynamics

## Abstract

It has recently become appreciated that cells self-organize their interiors through the formation of biomolecular condensates. These condensates, typically formed through liquid–liquid phase separation of proteins, nucleic acids, and other biopolymers, exhibit reversible assembly/disassembly in response to changing conditions. Condensates play many functional roles, aiding in biochemical reactions, signal transduction, and sequestration of certain components. Ultimately, these functions depend on the physical properties of condensates, which are encoded in the microscopic features of the constituent biomolecules. In general, the mapping from microscopic features to macroscopic properties is complex, but it is known that near a critical point, macroscopic properties follow power laws with only a small number of parameters, making it easier to identify underlying principles. How far does this critical region extend for biomolecular condensates and what principles govern condensate properties in the critical regime? Using coarse-grained molecular-dynamics simulations of a representative class of biomolecular condensates, we found that the critical regime can be wide enough to cover the full physiological range of temperatures. Within this critical regime, we identified that polymer sequence influences surface tension predominately via shifting the critical temperature. Finally, we show that condensate surface tension over a wide range of temperatures can be calculated from the critical temperature and a single measurement of the interface width.

## Significance

Self-organization through the phase separation of biomolecular condensates is ubiquitous in living cells. The macroscopic physical properties of these condensates play an essential role in their organization and function—what general principles relate these macroscopic properties to the underlying microscopic features of biomolecules? By using universal ratios of thermodynamic quantities in the vicinity of a critical point, condensate physical properties can be inferred from a small number of thermodynamic parameters, which we demonstrate in the case of surface tension. Importantly, we confirm via simulation that the range of validity of the critical region is large enough to cover the physiologically relevant range in living cells. This simplification allows us to identify key features of biomolecules that influence surface tension.

Living cells must exert both spatial and temporal control of their internal components to successfully carry out desired tasks. One ubiquitous mode of control is compartmentalization of internal components through the formation of biomolecular condensates (1). Canonical examples biomolecular condensates include P granules (2), stress granules (3), and nucleoli (4). These condensates are understood to form through liquid–liquid phase separation of biopolymers, such as proteins and nucleic acids, which form a scaffold for other molecules to colocalize in the condensates (5–8). Biomolecular condensates possess liquid characteristics such as fusion, flow, and continuous material exchange between dense and dilute phases, leading to functional advantages such as reversible response to environmental conditions, rapid internal mixing of components, and ready recruitment of reactants necessary for biochemistry or signaling (2, 4, 9–11). Ultimately, these macroscopic properties of the condensates are encoded in the microscopic features of the constituent biopolymers. Is there a systematic way to bridge between macro and microscales? We explore this question by focusing on the surface tension—a key macroscopic property of condensates that arises from the microscopic interactions among the component biopolymers (12).

Surface tension plays an important role in determining the equilibrium properties of condensates, influencing both their morphology and internal organization (13). For example, the relative surface tension between various bulk phases governs the hierarchical structure in multiphasic condensates (14–16). Furthermore, surface tension also influences nonequilibrium behavior, such as the rate of Ostwald ripening or the dynamics of fusion events (17, 18). A common experimental method of measuring surface tension involves a fusion assay to quantify the ratio between the viscosity and surface tension, combined with fluorescence recovery after photobleaching experiment to measure viscosity (2, 19). Such experiments have revealed that biomolecular condensates typically have extremely low surface tensions, ~10^−7^ to 10^−5^ N/m, about ~10^3^ times smaller than conventional phase separated systems such as oil and water (13). Low surface tension leads to relatively slow coarsening and easy deformation or fission within cells (20–22), but predicting which molecular features influence condensate surface tension has remained challenging.

In general, surface tension arises from the interactions between the molecules that constitute a condensate as well as their interactions with the solvent. For biomolecular condensates, these interactions are dependent on numerous factors, such as polymer sequence/valence, component stoichiometry, temperature, salt concentration, and pH (23–29). Ideally, in order to identify principles governing the surface tension, and other macroscopic condensate properties, we would like to reduce these complex microscopic details to a few physically relevant parameters. Parameter reduction can be achieved sufficiently close to criticality, where thermodynamic variables obey a power law with respect to the reduced temperature $\tau =1-T/T_{c}$ , where $T_{c}$ is the critical temperature (30). But does the critical regime, where power laws are valid, extend far enough to be biologically relevant? Previous experimental studies of synthetic polymers, such as polystyrene and polyisoprene, report the range of the critical regime to be 5 to 30 K from $T_{c}$ , with the exact range depending on system specific factors such as the degree of polymerization (31–33). However, biomolecular condensates are typically “network liquids”, held together by specific and saturating bonds between domains, and thus qualitatively different from synthetic polymer systems (34).

Typically, the range of the critical regime is thought to be comparable to $T_{\Theta }-T_{c}$ , where $T_{\Theta }$ is the theta temperature of the polymer solution, namely the temperature where ideal polymer behavior is observed (35). Of particular relevance to biomolecular condensates, $T_{\Theta }$ is expected to be substantially higher than $T_{c}$ for polymers that form specific bonds, due to their tendency to self-collapse (36). Furthermore, theory for the critical-to-mean-field cross-over predicts critical behavior up to a reduced temperature of $\tau \approx 1/\sqrt{N}$ , where N is the degree of polymerization of binding domains (37). These factors imply that the critical regime may be particularly large for biomolecular condensates. We therefore sought to directly quantify the range of the critical regime using a model system that captures the network-liquid character of such condensates. Specifically, we modeled systems of polymers that phase separate due to heterotypic, short-ranged, and saturating associations between interacting domains (“stickers”). We utilized the polymer sequence as a biologically relevant microscopic tuning parameter, as $T_{c}$ can be adjusted by solely changing the sequence of stickers in this model system, allowing us to compare systems with varying $T_{c}$ values on an equal footing.

Using molecular-dynamics simulations of the model system, we found that the range of the critical regime is about 20 to 25% of $T_{c}$ . Importantly, for condensates with $T_{c}\approx 300–400\;K$ typically expected for physiological condensates (38–41), this corresponds to the critical regime extending ~60 to 100 K below $T_{c}$ , which in many cases is sufficiently wide enough to span the physiologically relevant range of temperatures. We then demonstrated the utility of parameter reduction in the critical regime by showing that surface tension can be accurately estimated solely from $T_{c}$ and one value of the interface width between bulk phases. Overall, these results suggest that the framework of critical phenomena can be utilized as a principled approach to understand the effect of microscopic features on the macroscopic properties of many biomolecular condensates.

## Results

### Model System.

**Fig. 1.**
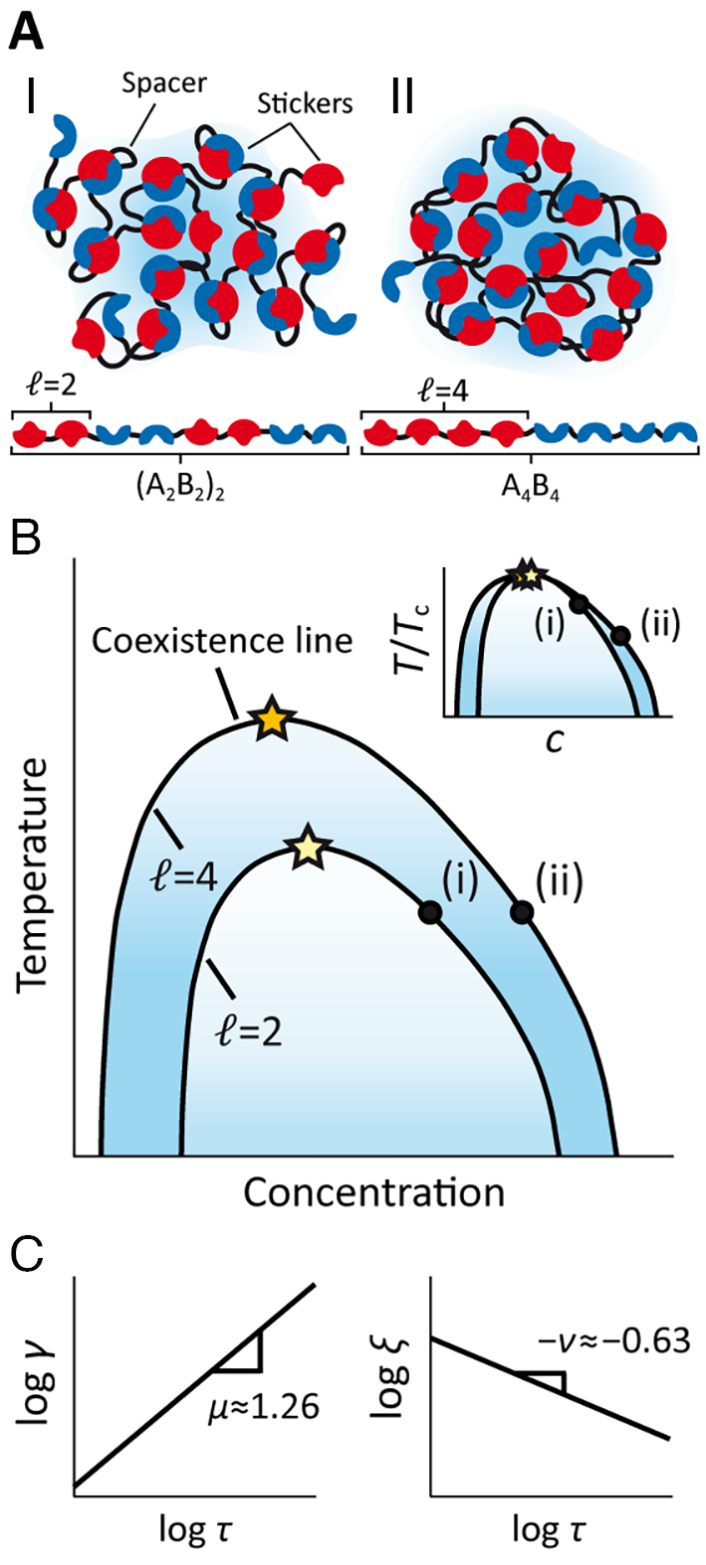
Phases and critical behavior of sticker and spacer associative polymers. (*A*) Multivalent interactions between polymers consisting of associative domains (stickers) connected by linkers (spacers) drive phase separation into condensates. Each polymer is composed of periodic repeats of *ℓ* stickers, where A and B denote the sticker types**.** Condensates composed of polymers with a block length of (i) *ℓ* = 2 and (ii) *ℓ* = 4 are illustrated. (*B*) Schematic representation of phase diagrams in the temperature-concentration plane. Block length influences the coexistence curves, leading to systems at the same absolute temperature (black dots) having different reduced temperatures $\tau =1-T/T_{c}$ (*Inset*), where $T_{c}$ is the critical temperature (stars). (*C*) Sketch of the expected power-law dependences on reduced temperature of (*Left*) surface tension and (*Right*) correlation length, with the corresponding critical exponents.

A sticker-spacer model of associating polymers was utilized to study the phase behaviors of two-component multivalent systems (42). Polymers were modeled as linear chains of spherical beads connected by stretchable springs, where each bead represents an associative sticker. Each polymer was composed of type “A” and “B” stickers that can form a specific and saturating heterotypic bond. To isolate the effects of polymer sequence, we focused on polymers comprised of periodic repeats of *ℓ* A stickers, immediately followed by *ℓ* B stickers (Fig. 1*A*), allowing us to parametrize polymer sequence via the block length *ℓ*. Furthermore, by choosing the degree of polymerization to be an integer multiple of 2*ℓ*, we ensured equal component stoichiometry between A and B stickers.

**Fig. 2.**
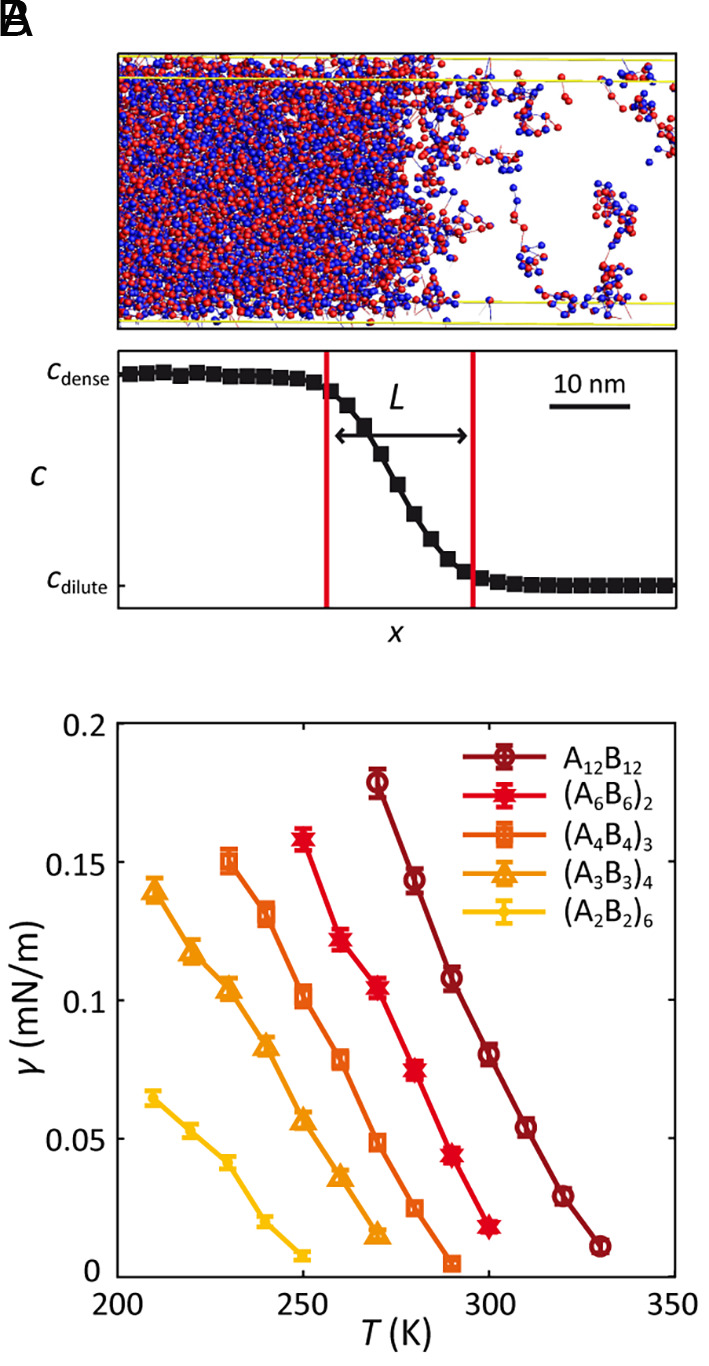
Coarse-grained molecular-dynamics simulations of associative polymers. (*A*) (*Top*) Snapshot of simulation at the interface of a system with 625 polymers of length 24, *ℓ* = 3, and *T* = 260 K in a 250-nm × 30-nm × 30-nm box. Each polymer was modeled as linear chain of spherical particles connected by stretchable bonds, where sticker A is shown in red and B in blue. (*Bottom*) Monomer concentration profile along the axis perpendicular to the interface and the corresponding interface boundaries (red) are shown, where *L* denotes the interface width (see text). (*B*) Surface tension dependence on block length. Surface tension versus absolute temperature for systems with length 24 polymers with block lengths ranging from *ℓ* = 2 to 12 was calculated using Eq. **9** and averaged over time and over five simulation repeats. Error bars are SEM.

We performed coarse-grained molecular-dynamics simulations using Large-scale Atomic/Molecular Massively Parallel Simulator (LAMMPS) to obtain the thermodynamic properties of the model systems (43). To enforce one-to-one heterotypic interaction between stickers, we imposed an attractive interaction between stickers of the two different types, A and B, and a stronger repulsive interaction between stickers of the same type. Molecular-dynamics simulations were performed using Langevin dynamics in a periodic simulation box with fixed dimensions and number of polymers (Fig. 2*A*). See *Materials and Methods* for details.

### Range of the Critical Regime.

Near criticality, the relevant parameter that characterizes thermodynamic variables is the reduced temperature $\tau =1-T/T_{c}$ , which is a dimensionless measure of how far the system is from the critical point (Fig. 1*B*). In this regime, thermodynamic variables display a simple power-law behavior with respect to τ (Fig. 1*C*). For example, the power law exhibited by surface tension is

[1] $$\gamma =\gamma_{0}\tau^{\mu },$$

where $\gamma_{0}$ is the critical amplitude for surface tension, and μ≈1.26 is the critical exponent for surface tension in the three-dimensional Ising universality class (44, 45). In general, critical exponents are independent of many system details, and have universal values within each universality class, which is determined by the dimensionality and the symmetries of the system and the dimension of the order parameter (30). Polymer solutions are described by the Ising universality class, due to the $Z_{2}$ symmetry of the scalar order parameter, namely density (46). Note that systems with an upper critical solution temperature (UCST) or a lower critical solution temperature (LCST) can be part of the Ising universality class. Whether a system has a UCST or an LCST is determined by how the interaction parameter scales with temperature—if the strength of attraction (in thermal units) decreases with increasing temperature, the system will have a UCST, if the strength of attraction increases with temperature, the system will have an LCST.

**Fig. 3.**
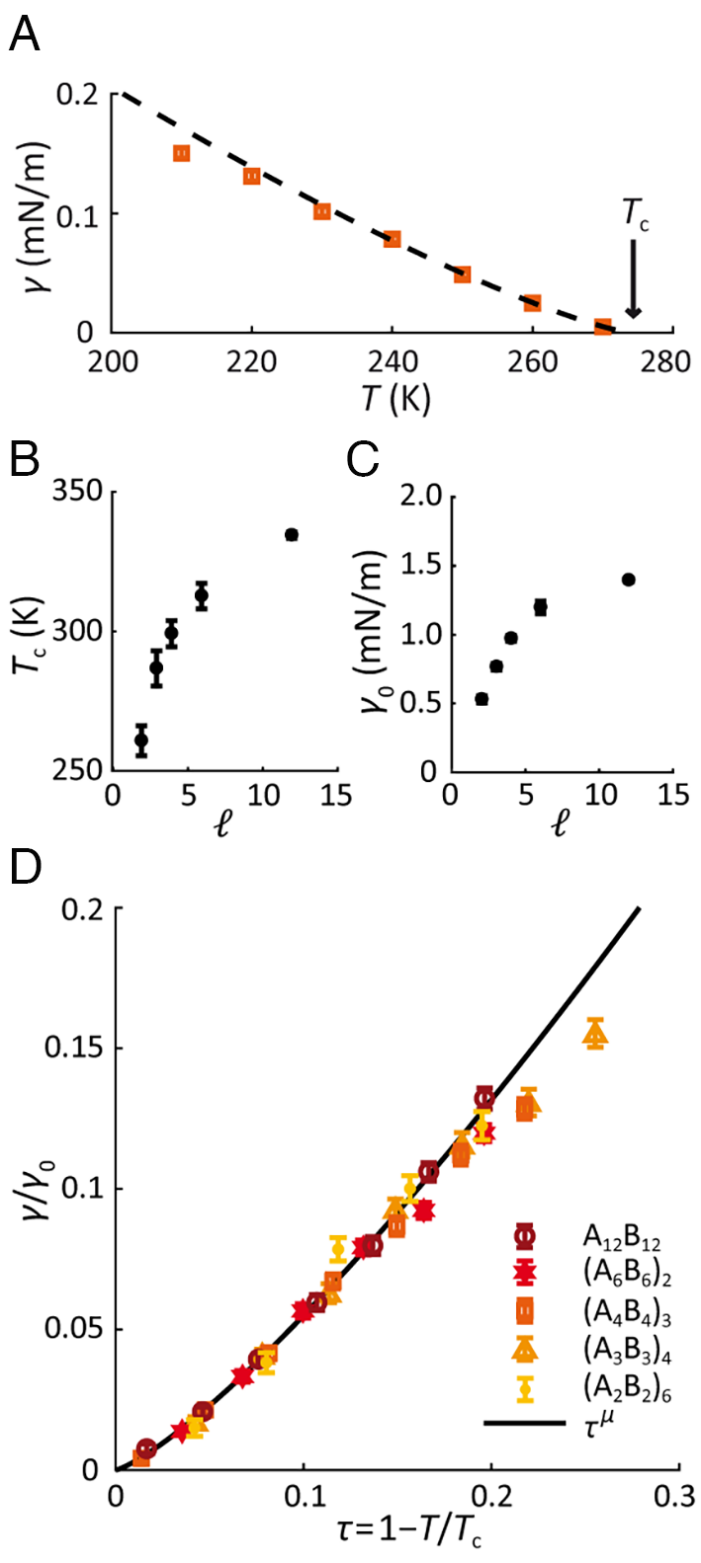
Power-law behavior of surface tension near the critical point. (*A*) Surface tension obtained from simulations at different temperatures was fit to the expected power law (Eq. **1**) to determine the critical temperature $T_{c}$ and the critical amplitude $\gamma_{0}$ for polymer systems with varying block lengths *ℓ*. Example shown for *ℓ* = 4. (*B*) Critical temperature and (*C*) critical amplitude obtained from fit for block lengths ranging from *ℓ* = 2 to 12. (*D*) Surface tension values scaled by critical amplitude $\gamma_{0}$ collapse onto a universal curve with respect to reduced temperature τ . Error bars are SEM.

Does the critical regime, where power laws are valid, extend far enough to be biologically relevant? In order to make quantitative estimates of the range of the critical regime, we opted for direct estimation via coarse-grained molecular-dynamics simulation. Briefly, surface tension over a range of temperatures was directly calculated from simulations using the Kirkwood–Buff formula (47) (*Materials and Methods*). The resulting temperature profile of surface tension was then fit to Eq. **1** (Fig. 3*A*), where $\gamma_{0}$ and $T_{c}$ were extracted as fitting parameters for each polymer system with given l (Fig. 3 *B* and *C*). As seen in Fig. 3*D*, plotting the scaled surface tension $\gamma /\gamma_{0}$ with respect to the reduced temperature revealed that for all the systems studied the surface tension accurately obeys the pure power-law behavior in Eq. **1** until τ≈0.2 or even beyond. Noting that biological condensates typically have critical temperatures in the physiological regime, above ~300 K, a critical regime extending to τ≈0.2 implies that the power laws hold ≳60 K below the critical temperature. This suggests that for biomolecular condensates, the influence of the critical regime may extend over the entire physiological range of temperatures.

### Effect of Polymer Block Length on Surface Tension.

According to Eq. **1**, the surface tension in the critical regime depends both on the critical amplitude and on reduced temperature τ . As shown in Fig. 2*B*, direct calculation of the surface tension from simulations reveals that systems of polymers with longer l have higher surface tension at any given temperature. How does l influence both $\gamma_{0}$ and $T_{c}$ to yield the observed ordering of surface tension? Considering the critical temperature for each system (Fig. 3*B*), we found that $T_{c}$ monotonically increases with l , varying by approximately 80 K between the l=2 and l=12 . On the other hand, $\gamma_{0}$ varies by at most a factor of 3, and typically much less (Fig. 3*C*). Due to the functional form of surface tension in Eq. **1**, the change in τ as a result of the shift in $T_{c}$ is the dominant factor—for example, the $\gamma_{0}$ values for the l=6 and l=12 systems differ only by 15%, meaning that the 350% difference in their surface tensions at *T* = 300 K arises almost entirely from the difference of about 20 K in their critical temperatures. [Mechanistically, the increase in $T_{c}$ for polymers with larger l is a consequence of their lower conformational entropy in the dilute phase (48).] Thus, in general, the observed block-length dependence of surface tension essentially reflects differences in $T_{c}$ , leading us to conclude that in these model systems proximity to criticality is the key determinant of surface tension.

### Thermodynamic Variables in the Critical Regime are Interdependent.

The existence of power-law behavior in the critical regime provides a universal method of parameterization, whereby any thermodynamic variable can be described in terms of $T_{c}$ and a specific critical amplitude. We wondered if further reduction in the number of relevant parameters was possible. To this end, we utilized an expected universal ratio between thermodynamic variables to achieve further parameter reduction. Universal ratios of thermodynamic variables arise as a consequence of the two-scale-factor universality hypothesis, which in short states that only two thermodynamic scales are required to fully specify the universal correlation function (49). This implies that within a universality class, every thermodynamic variable is fully specified if two independent critical amplitudes and $T_{c}$ are known. For our modeled biomolecular condensates, we decided to test the universal ratio $R_{-}$ between surface tension and correlation length (50), given by

[2] $$R_{-}=\frac{\gamma \xi^{d-1}}{k_{B}T_{c}}\approx 0.1024,$$

where $k_{B}$ is the Boltzmann constant, d=3 is the dimensionality of the system, and ξ is the correlation length, which in the critical region obeys

[3] $$\xi =\xi_{0}\tau^{-\nu },$$

with ν=μ/(d-1)≈0.63 for the 3D Ising model class (44). There are other universal ratios not explored in this work, which can also be used to relate physical properties, such as compressibility, heat capacity, capillary length, and the concentration difference between bulk phases (51, 52).

In practice, the correlation length is not a convenient quantity to obtain from simulations or experiments in the two-phase regime. Instead, from the Ornstein–Zernike theory of correlation functions, ξ can be shown to be proportional to the interface width L (44). Therefore, we obtained the interface width from simulations by fitting the concentration profile at the interface to the expression

[4] $$c\left(x\right)=\frac{1}{2}\left(c_{1}+c_{2}\right)+\frac{1}{2}\left(c_{2}-c_{1}\right)\tanh\frac{2\left(x-x_{0}\right)}{L},$$

where $c_{1}$ and $c_{2}$ are dilute and dense phase concentrations respectively, $x_{0}$ is the midpoint of the interface, and L is the interface width (Fig. 2*A*). The correlation length can be related to the decay rate of the density profile asymptotically far from the interface (*Materials and Methods*), yielding the relation L=4ξ . Using this relation in Eq. **2** then yields

**Fig. 4.**
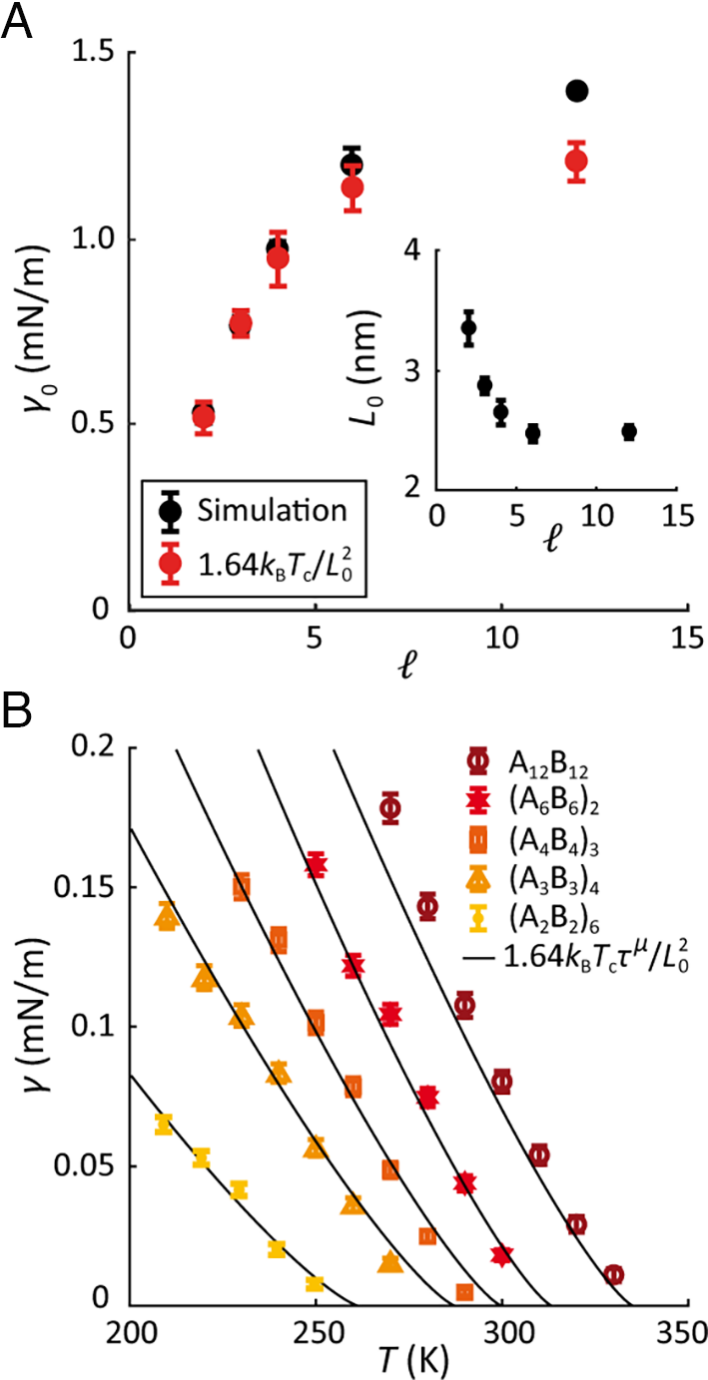
Surface tension over a large range of T is a simple function of critical temperature $T_{c}$ and the interface width critical amplitude $L_{0}$ . (*A*) Critical amplitudes $\gamma_{0}$ for the surface tension (black dots), and $\gamma_{0}$ estimated from the critical temperatures and the critical amplitudes $L_{0}$ for the interface width using Eq. **5** (red dots) are shown. (*Inset*) Critical amplitude for interface width for polymers with different block lengths *ℓ* determined from a fit of simulation density profile to Eq. **3**, and corresponding scaling law. (*B*) Comparison of surface tension versus temperature from simulations (data points) with theoretical predictions (black curves). Error bars are SEM.

[5] $$\gamma \approx 1.64\frac{k_{B}T_{c}}{L^{2}},$$

which can be used to estimate surface tension from the critical temperature and the interface width. Moreover, since L exhibits the same power-law behavior as ξ (*SI Appendix*, Fig. S7), one only needs to know $L_{0}$ (or equivalently the value of L anywhere in the critical regime) to calculate the temperature profile of surface tension. As seen in Fig. 4*A*, we find that the critical amplitude $\gamma_{0}$ for surface tension calculated using Eq. **5** agrees well with the simulation results. Thus, the full temperature-dependent surface tension can be recovered from $T_{c}$ and one measurement of the interface width (Fig. 4*B*). [Note that the apparent width of the interface may be widened by capillary waves; we verified that our measured values of L are not overly affected by capillary waves by comparing the correlation lengths inferred from the interface width with correlation lengths directly calculated from bulk-phase density fluctuations far from the interface (*SI Appendix*)].

## Discussion

In biomolecular condensates, macroscopic properties emerge from the microscopic features of the constituent biopolymers. However, the complex mapping across scales makes it difficult to infer the influence of a given microscopic feature on macroscopic physical properties. In this work, we illustrated how to bridge these disparate scales using the tools of critical phenomena. We specifically focused on condensate surface tension as a biologically important but generally hard to predict macroscopic property. In general, the range of temperatures over which critical scaling is valid depends on system specific details and is difficult to predict. Therefore, we employed coarse-grained molecular-dynamics simulations to directly determine the critical regime and found that for biomolecular condensates the critical regime can be large enough to encompass the full physiological range (~25% of *T*_c_). From these simulations, we found that the magnitude of surface tension varies dramatically for different polymer sequences, and that this variation is almost entirely due to shifts in *T*_c_. Furthermore, to demonstrate that the interdependence of thermodynamic quantities in the critical regime is applicable to biomolecular condensates, we showed that the full surface tension versus temperature relation can be accurately inferred from the critical temperature and one measurement of the interface width.

Many natural and engineered condensates are held together by specific one-to-one interactions between domains. Examples include the algal pyrenoid, composed of the enzyme Rubisco and linker protein EPYC1, and engineered condensates such as the SIM-SUMO and SH3-PRM systems (34, 53). To simulate such biomolecular condensates, we employed an idealized model of polymers with sticker–sticker interactions, along with an implicit solvent. Specifically, one-to-one association between stickers of different types was implemented by allowing the two stickers to overlap in space (54). Although such complete overlap is somewhat unphysical, the low concentration of the dense phase (<10% volume fraction) means that this overlap has essentially no effect on equilibrium properties. Moreover, the only nonspecific interaction implemented in the model system was volume exclusion between stickers of the same type. However, in real condensates, nonspecific interactions may influence the phase properties as well—how might attractive nonspecific interactions modulate the extent of the critical region? At one extreme, phase separation driven solely by nonspecific interactions, such as in a Lennard–Jones polymer solution, has an even wider critical regime (up to τ≈0.4 ) (55, 56). We found that addition of a weak Lennard–Jones interaction to our model system, alongside the specific sticker–sticker interactions, still preserved a large critical regime, τ≈0.2 , along with the expected universal relationship between surface tension and interface width (Eq. **5**) (*SI Appendix*, Fig. S2). An important question is whether our results still apply in the context of living cells, which are typically out of equilibrium, and spatially heterogeneous. One reason to expect our results also apply in vivo is that the nonequilibrium processes in cells that alter local concentrations of biomolecules or modify their interactions typically operate on much longer timescales (on the order of minutes or more) (7, 53, 57) than the equilibration timescale of phase separating proteins and RNAs (typically on the order of seconds or less) (19, 58), implying that equilibrium thermodynamics govern instantaneous properties, as demonstrated for P granules in *Caenorhabditis* *elegans* (59). Moreover, condensates reconstituted in vitro at equilibrium typically display physical properties such as surface tension and internal remixing times consistent with their in vivo counterparts (13, 14), supporting the above collusion based on separation of time scales. Thus, despite the simplicity of our model, we expect our finding of a large critical regime will extend to a broad range of biomolecular condensates.

In addition to the simplification associated with the universal power-law behavior of thermodynamic properties in the critical regime, critical amplitudes (i.e. prefactors) are also interrelated via the two-scale-factor universality hypothesis. The universal ratio that relates surface tension to correlation length was utilized in this work (Eq. **2**), where the relationship was expressed in terms of interface width L instead of correlation length ξ . The resulting expression for surface tension (Eq. **5**) resembles the usual estimate for the surface tension of simple fluids, $\gamma ∼E/a^{2}$ , where E is a typical energy scale, and a denotes the particle size (60). However, unlike in simple fluids, “particle size” is ambiguous for polymeric systems where there are multiple length scales such as monomer size, average linker length between monomers, radius of gyration, etc. As implied in Eq. **5**, we find that the appropriate length scale in the critical regime is the interface width L , and the appropriate energy scale is $k_{B}T_{c}$ . Furthermore, substituting typical values of surface tension for biomolecular condensates (~10^−7^ to 10^−6^ N/m) into Eq. **5**, we predict the interface width to be on the order of hundreds of nanometers, which would make it possible to measure L using advanced imaging techniques such as superresolution microscopy (61). Moreover, from L , both correlation length ξ and surface tension γ can be calculated, suggesting a passive method of measuring surface tension in vivo.

For phase separating systems driven by strongly temperature-dependent interactions such as hydrophobicity, the values of the underlying interaction parameters at criticality may substantially differ from their values away from the critical point. In order to apply the tools of critical phenomena to such systems, an effective $T_{c}$ calculated by assuming temperature-independent parameter values can be utilized in lieu of the true $T_{c}$ . A more serious complication is that in biological systems changes in temperature can trigger adaptive responses such as the heat shock response, which can alter both the constituents and the environmental conditions of condensates. For in vivo studies, one way of avoiding the difficulties associated with temperature change is to recast the critical framework in terms of variables that can be more practically independently modulated, such as the relative or absolute concentrations of phase separating components. In the case of complex coacervates, power laws constructed with respect to variables other than temperature have provided significant insight into the relation between microscopic and macroscopic properties. For example, power-law dependence of surface tension on microscopic properties such as degree of polymerization and strength of electrostatic interactions has been found to agree quantitatively with experiments (62, 63). Investigating the critical properties with respect to alternate variables in place of temperature will be useful in practical applications, and is a topic of future study.

The universality of behavior near a critical point provides an inherently principled way to relate microscopic features to macroscopic properties. Within a model for biomolecular phase separation, this simplification allowed us to infer that polymer sequence primarily influences surface tension by shifting the critical temperature, thereby changing the effective distance to the critical point. Universal behavior of thermodynamic properties in the critical regime also allowed us to relate surface tension, correlation length, and interface width. More broadly, this interdependence makes it possible to calculate thermodynamic variables in terms of experimentally accessible observables. Notably, the scaling laws that describe thermodynamic properties, as well as their interdependence are not limited to a particular model system and are generally applicable to phase separating systems within the 3D Ising universality class. For example, universal amplitude ratios (e.g., Eq. **2**) have been found to be consistent with computational and experimental studies for a range of fluids, regardless of the nature of the interactions. We thus anticipate that thermodynamic relations will apply to a broad range of biomolecular condensates.

## Materials and Methods

### Simulation Details.

#### Interaction model.

Coarse-grained molecular-dynamics simulations were performed using LAMMPS (43). Two-component associative polymers were modeled as a linear chain of spherical beads of diameter d=1 nm , where each bead represents a sticker domain. The beads were connected by stretchable bonds given by

[6] $$U_{b}=-\frac{1}{2}KR_{0}^{2}\log\left(1-\frac{r^{2}}{R_{0}^{2}}\right),$$

where $K=0.56\;k_{B}T/nm^{2},\;and\;R_{0}=5\;nm$ . Stickers of different types were chosen to interact through an attractive potential given by

[7] $$U_{\text{a}}=\left\{\begin{array}{c} - \end{array}\begin{array}{ll} \frac{1}{2}U_{0}\left[1+\cos\left(\frac{\pi r}{d}\right)\right], & r<d\\ 0, & r\geq d \end{array}\right.,$$

where $U_{0}=8k_{B}T_{0}$ with $T_{0}=300\;K$ . One-to-one binding of the stickers of the same type was enforced by including a purely repulsive Lennard–Jones potential

[8] $$U_{r}=\left\{\begin{array}{c} 4\varepsilon \left[{\left(\frac{d}{r}\right)}^{12}-{\left(\frac{d}{r}\right)}^{6}+\frac{1}{4}\right],\;\\ 0,\;\;\;\;\;\;\;\;\;\;\;\;\;\;\;\;\;\;\;\;\;\;\;\;\;\;\;\;\;\;\;\;\;\;\;\; \end{array}\right.\begin{array}{c} r<2^{1/6}d\\ r\geq 2^{1/6}d \end{array},$$

where $\varepsilon =k_{B}T_{0}$ . Values of interaction parameters were chosen to yield a radius of gyration, $R_{g}$ , in the range of 2 to 4 nm (*SI Appendix*), which is comparable to $R_{g}$ of the prion-like domain of heterogeneous nuclear ribonucleoprotein A1, which 19 associative sites, similar to the 24 stickers per polymer employed in the current study (64).

#### Simulation procedure.

Periodic repeat polymers with degree of polymerization of 24 and equal A:B stoichiometry were simulated in a periodic simulation box of dimensions 250 nm×30 nm×30 nm , using a timestep of $\tau_{v}/100$ where $\tau_{v}$ is the velocity relaxation time of the beads. The mass of each bead, m , was set such that $\tau_{v}=mD/k_{B}T=1\;ns$ . In order to promote the formation of a single condensate, 625 polymers were initialized in a slab geometry (65) (confined to ±40 nm ) and allowed to equilibrate with no associative interaction ( $U_{0}=0$ ) for $10^{6}$ timesteps. The associative interaction $U_{a}\left(r\right)$ was then gradually switched on from $U_{0}=0$ to $U_{0}=8k_{B}T$ over $10^{6}$ timesteps, at which point the polymers were no longer confined to the slab and allowed to equilibrate for another $10^{7}$ timesteps (*SI Appendix*, Fig. S1). After equilibration, five independent simulation runs of ${5\times 10}^{7}$ timesteps were made.

### Computational Details.

#### Surface tension.

Surface tension was calculated directly from simulations using the Kirkwood–Buff formula (47), given by

[9] $$\gamma =\frac{L_{x}}{2}\langle p_{x}-\frac{1}{2}\left(p_{y}+p_{z}\right)\rangle ,$$

where $L_{x}=250\;nm$ is the length of the long axis of the simulation box, and $p_{j}$ is the diagonal entry in the pressure tensor along the j th axis extracted every $10^{5}$ timesteps. The factor of ½ in Eq. **9** arises from the existence of two interfaces in the slab geometry.

#### Interface width and correlation length.

First, the number density profile along the *x*-axis of the simulation box was determined by binning the position of the monomers every $10^{5}$ timesteps (500 time slices per simulation). Then, the interface width was determined by fitting the number density profile to Eq. **4**. Next, correlation length was calculated from the interface width using an expression from the Ornstein–Zernike theory of correlation functions for effective one-dimensional concentration profile

[10] $$\lim_{x\to \infty }c\left(-x\right)-c_{1}=c_{2}-\lim_{x\to \infty }c\left(x\right)\sim e^{-x/\xi },$$

where $c_{1}$ and $c_{2}$ are dilute and dense phase concentrations, respectively (44). Substituting Eq. **4** into Eq. **10** yields the desired relation between the interface width and correlation length, namely L=4ξ.

#### Critical temperature and amplitudes.

The critical temperature and critical amplitude for surface tension was determined by fitting the temperature profile of surface tension to Eq. **1**. The critical amplitude for correlation length was determined by fitting L/4 calculated from simulations to Eq. **3**, along with the associated $T_{c}$ . $T_{c}$ calculated from surface tension agreed with the $T_{c}$ determined independently from coexistence density profiles (*SI Appendix*). In order to avoid the influence of finite size effects in determining the critical temperature and amplitudes, the simulation at a given temperature was only utilized in the fit if the implied correlation length was $<0.05L_{x}$.

## Supplementary Material

Appendix 01 (PDF)

## Data Availability

MatLab code to generate LAMMPS simulation codes, LAMMPS simulation codes, LAMMPS simulation data, and MatLab analysis codes have been deposited in agpyo/matlab-lammps-stickersims (https://github.com/agpyo/matlab-lammps-stickersims) (66).
