# Supplementary material for: Proximity to criticality predicts surface properties of biomolecular condensates
Source: Proc Natl Acad Sci U S A. 2023 May 30;120(23):e2220014120. doi: 10.1073/pnas.2220014120 (PMC10266063; doi:10.1073/pnas.2220014120)
Supplement: Supplementary file 1 — Appendix 01 (PDF) [file pnas.2220014120.sapp.pdf]

PNAS

Supporting Information for
Proximity to Criticality Predicts Surface Properties of Biomolecular Condensates

Andrew G. T. Pyo, Yaojun Zhang, and Ned S. Wingreen

Corresponding Authors: Yaojun Zhang (yaojunz@jhu.edu) and Ned S. Wingreen (wingreen@princeton.edu)

This PDF file includes:

Supporting text
Figures S1 to S7
SI References

Supporting Information Text

1. Simulation with specific and non-specific associative interactions. An additional non-specific associative interaction was implemented in the coarse-grained molecular dynamics simulation by extending the range of the Lennard-Jones potential between stickers. As before, polymers were modeled as a linear chains of spherical beads of diameter $\boldsymbol{d} = \mathbf{1}$ nm, where each bead represents a sticker domain. The beads were polymerized via a stretchable bond with energy

$$U_{\mathrm{b}} = -\frac{1}{2}KR_0^2 \log\left(1 - \frac{r^2}{R_0^2}\right), \quad \text{[S1]}$$

where $K = 0.56k_{\mathrm{B}}T/\mathrm{nm}^2$, $R_0 = 5$ nm, and r is the center-to-center distance between beads. The specific interaction between stickers of different types was enforced by an attractive potential given by

$$U_{\mathrm{a}} = \begin{cases} -\frac{1}{2}U_0\left[1 + \cos\left(\frac{2\pi r}{d}\right)\right], & r < d \\ 0, & r \geq d' \end{cases} \quad \text{[S2]}$$

where $U_0 = 8k_BT_0$ with $T_0 = 300$ K. A Lennard-Jones non-specific interaction between beads of the same type was imposed by replacing the purely repulsive interaction (U_{r}) with the partially attractive interaction

$$U_{\mathrm{ns}} = \begin{cases} 4\varepsilon\left[\left(\frac{d}{r}\right)^{12} - \left(\frac{d}{r}\right)^6 + c\right], & r < 3(2^{1/6})d \\ 0, & r \geq 3(2^{1/6})d' \end{cases} \quad \text{[S3]}$$

where $\varepsilon = 0.03U_0$, and $c = \frac{1}{2}(3d)^{-6}\left[1 - \frac{1}{2}(3d)^{-6}\right]$.

Similar to the simulation procedure described in the main text, 625 $(A_4B_4)_3$ polymers were simulated in box of dimensions 250 nm × 30 nm × 30 nm, using a timestep of $\tau_v/100$ = 0.01 ns. Polymers were initialized in a slab geometry and confined to a region of size 80 nm × 30 nm × 30 nm central to the simulation box and allowed to equilibrate with $U_0 = 0$ for 10^6 timesteps. U_0 was then gradually increased to reach the final value of $U_0 = 8k_BT_0$ over 10^6 timesteps, then the system was allowed to equilibrate without confinement for another 10^7 timesteps. After equilibration, 5 independent simulation runs of 5×10^7 timesteps were made from which surface tensions and interface widths were calculated.

From the simulations described above, surface tension was calculated as described in the main text. As seen in Fig. S2, the surface tension calculated from the simulations agrees with the expected power law,

$$\gamma = \gamma_0\left(1 - \frac{T}{T_{\mathrm{c}}}\right)^{\mu}, \quad \text{[S4]}$$

yielding fitting parameters $\gamma_0 = 3.7 \pm 0.3$ mN/m, and $T_{\mathrm{c}} = 373 \pm 2$ K (note the three-fold increase in γ_0, and a 25% increase in T_c compared to the equivalent simulation with purely repulsive non-specific interactions).

Following the main text, we utilized the simulations described above to verify the interdependence of surface tension and interface width. Specifically, the interface width L_0 and critical

temperature T_c were calculated from simulations as described in the main text. Then, employing the universal relation between surface tension and interface width, $\gamma_0 \approx 1.64\frac{k_B T_c}{L_0^2}$, we obtained an estimate of the critical amplitude for surface tension of $\gamma_0 = 3.1 \pm 0.6$ mN/m, which, within error, agrees with the value directly extracted from the fit to Eq. S4.

2. Critical temperature calculation. Typically, T_c is estimated by simultaneously fitting the law of rectilinear diameters,

$$\frac{\rho_1+\rho_2}{2} = \rho_c + A(T_c - T) \ , \qquad \text{[S5]}$$

and the universal power law for density differences between the two phases

$$\rho_2 - \rho_1 = \Delta\rho_0 \left(1 - \frac{T}{T_c}\right)^{\beta} \ , \qquad \text{[S6]}$$

where ρ_1 and ρ_2, respectively, are the dilute and dense phase number density of monomers, A is a fitting parameter, ρ_c is the critical number density, $\Delta\rho_0$ is the critical amplitude, and $\beta \approx 0.325$ is the expected critical exponent (1). As seen in Fig. S3, T_c values estimated from the co-existence densities as described above agree well with the estimates of T_c obtained from fitting the temperature dependence of the surface tension to Eq. S4. Similar results have been reported for Lennard-Jones polymer solutions (2).

3. Radius of gyration. The radius of gyration for each polymer was calculated using the equation

$$R_g^2 = \frac{1}{N}\sum_{n=1}^{N}(\boldsymbol{r}_n - \bar{\boldsymbol{r}})^2 \ , \qquad \text{[S7]}$$

where $N = 24$ is the degree of polymerization, r_n is the location of the nth sticker, and $\bar{\boldsymbol{r}} = \sum_{n=1}^{N}\boldsymbol{r}_n / N$ is the mean position of stickers. Polymers that were $2L$ away from the interface center were considered to be in a bulk phase where L is the measured interface width. The radii of gyration of polymers in the dense phase, and in the dilute phase are shown in Fig. S4.

4. Direct calculation of correlation length. To verify that our calculated interface widths were not dominated by capillary waves, correlation lengths were directly calculated using the density-fluctuation correlation function

$$g(\Delta x) \equiv \langle (c(x) - \bar{c})(c(x + \Delta x) - \bar{c}) \rangle \sim e^{-\frac{|\Delta x|}{\xi}} \ , \qquad \text{[S8]}$$

where x is the position along the axis perpendicular to the interface, $c(x)$ is the sticker concentration at position x, $\bar{c}$ is the average sticker concentration in the bulk phase, and ξ is the correlation length. Density fluctuations in the middle third of the dense phase were used to calculate the correlation length to avoid influence from the interface. The exponential behavior of the density fluctuation correlation function is shown in Fig. S5A. As seen in Fig. S5B, correlation lengths determined from Eq. S8 follow the power-law relation expected for the 3D Ising universality class ($\xi \sim \tau^{-0.63}$). Eq. 5 in the main text relates surface tension γ to the correlation length ξ as $\gamma \propto \xi^{-2}$. To determine the influence of capillary waves on our critical-scaling-based prediction for surface tension, the inverse square of the critical amplitude for the interface width L_0 directly calculated from the interface width L, and separately inferred from the correlation length using the relation $L = 4\xi$ are shown in Fig. S5C.

Although the value of L_0^{-2} calculated from the correlation length is systematically slightly higher, most values agree within our error bars, implying that the values of interface width L we obtained from simulations are only slightly increased by capillary waves (often within our simulation error bars). This confirms that the relation we found between surface tension and interface width based on critical scaling reflects the underlying physics of proximity to the critical point.

5. Finite-size effects. To verify that our simulation results are not dominated by finite-size effects, we performed additional simulations with varying box lengths L_x, as well as varying cross-sectional area A, following the same simulation steps as described in the text. Using the $(A_6B_6)_2$ system at $T = 280$ K to gauge the effect of finite size, we found that the surface tension is essentially independent of the box length around $L_x = 250$ nm (Fig. S6A), and cross-sectional area $A = 900$ nm^2 (Fig. S6B). This implies that our choice of box length in the main text ($L_x = 250$ nm), and the cross-sectional area ($A = 900$ nm^2) is sufficiently large to be unaffected by the finite size of the simulation box.

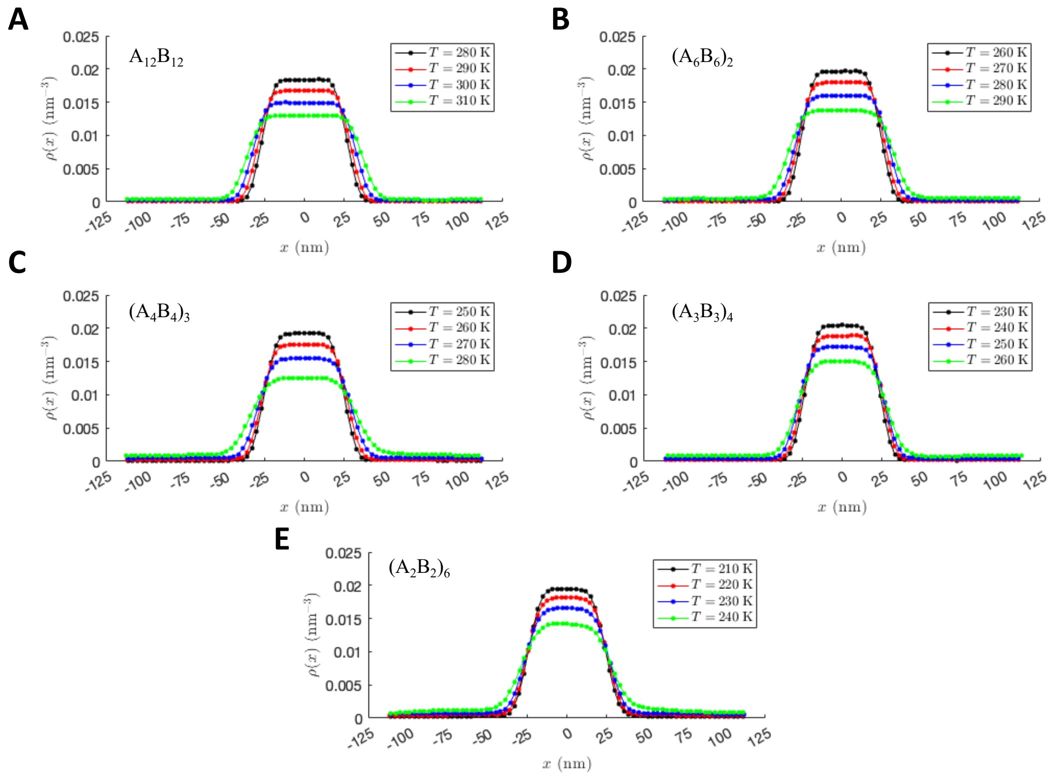

Fig. S1. Monomer number density profiles from associative polymer simulations. Monomer number densities $\rho(x)$ are shown along the axis perpendicular to the interface, calculated from an aligned average of 250 time slices over 5 simulations performed as in Fig. 2 of main text. Density profiles for simulations at reduced temperature within the range of $0.05 < 1 - T/T_c < 0.20$ are shown for systems composed of (A) $A_{12}B_{12}$, (B) $(A_6B_6)_2$, (C) $(A_4B_4)_3$, (D) $(A_3B_3)_4$, and (E) $(A_6B_6)_2$ polymers.

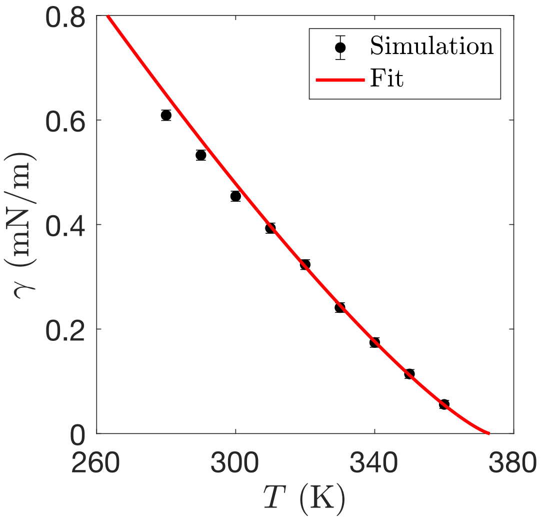

Fig. S2. Temperature dependence of surface tension for a phase-separated polymer system driven by both specific and non-specific associative interactions. Surface tension (black data points) is calculated from coarse-grained molecular-dynamics simulations of 625 $(A_4B_4)_3$ polymers. For simulation details see *SI Appendix 1*. Surface tension was calculated as described in the main text. Best-fit curve (red) to Eq. S4 of the first four datapoints closest to the critical temperature resulted in $\gamma_0 = 3.7 \pm 0.3$ mN/m, and $T_\mathrm{c} = 373 \pm 2$ K. Error bars indicate the standard error of the mean over 5 simulation runs.

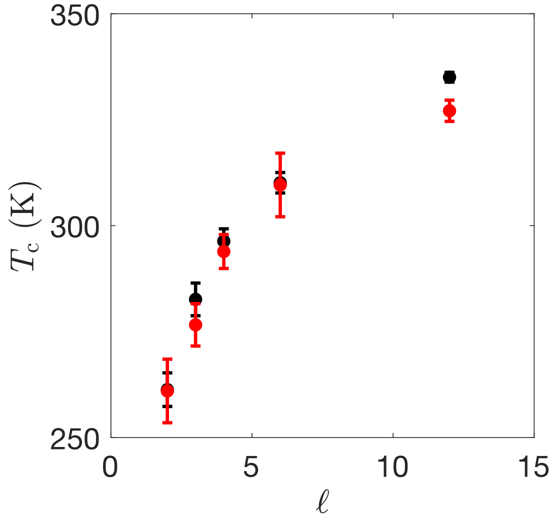

Fig. S3. Critical temperatures calculated from simulation. Critical temperature estimated from fit of surface tension to Eq. S4 (black), and from fit of co-existence densities to Eqs. S5 & S6 (red) are shown for polymer systems over a range of block lengths ℓ. Error bars for fits indicate the standard error of the mean over 5 simulation runs performed as in Fig. 2 of the main text.

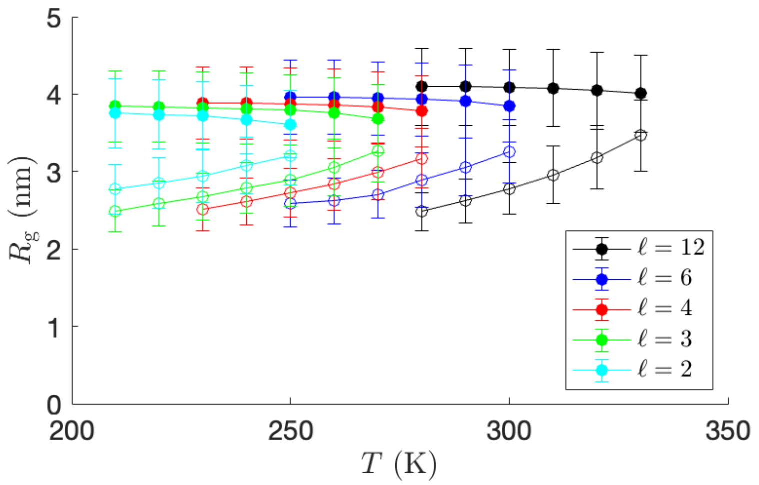

Fig. S4. Radii of gyration R_g of polymers of block length ℓ in the dense and dilute phases calculated from simulations performed as in Fig. 2 of the main text. Solid data points indicate R_g in the dense phase, while empty data points indicate R_g in the dilute phase.

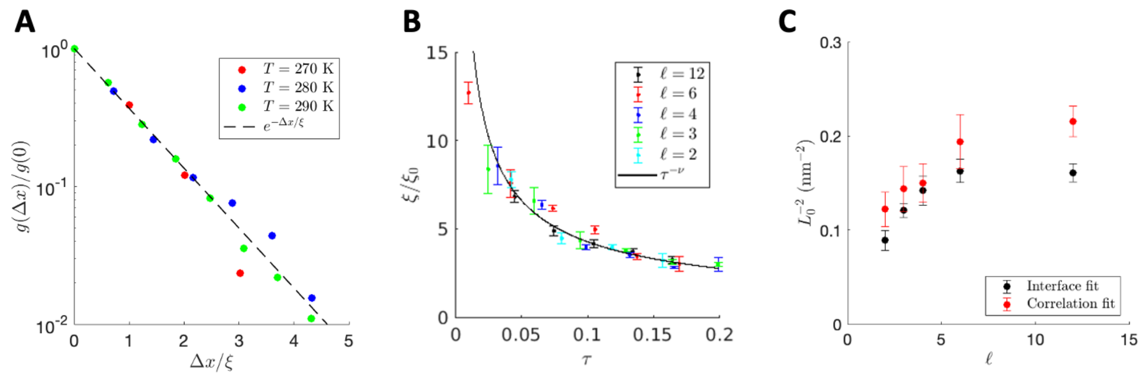

Fig. S5. Correlation length ξ and interface width L. (A) Density fluctuation correlation function calculated from simulations performed as in Fig. 2 of the main text for the $\ell = 6$ system at $T = 270$ K (red dots), $T = 280$ K (blue dots), and $T = 290$ K (green dots) display the expected exponential behavior from Eq. S8 (black dashed line). (B) Normalized correlation lengths calculated from the dense-phase density-fluctuation correlation function using Eq. S8 for block lengths from $\ell = 2$ to $\ell = 12$, and the power law expected for the 3D Ising universality class $\xi \sim \tau^{-0.63}$ (black curve) are shown as function of reduced temperature τ. (A) Inverse square of the interface width calculated from a fit to the interface density profile (black dots), and obtained from the correlation length using the relation $L = 4\xi$ (red dots), are shown. The error bars indicate the standard error of the mean.

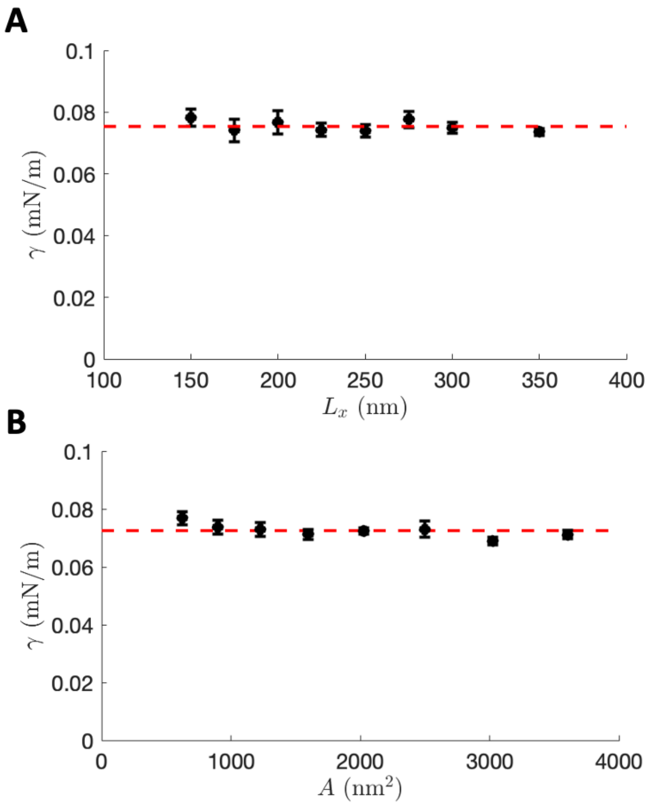

Fig. S6. Surface tension for varying simulation box dimensions. Surface tension was averaged over five independent simulation runs, performed as in Fig. 2 of the main text, of the $\ell = 6$ system at $T = 280$ K, for varying values of the simulation box length perpendicular to the interface (L_x) (A), and the square cross-sectional area (A) (B). The error bars indicate the standard error of the mean over simulation runs. In each case, the overall average value of surface tension across varying box dimensions is shown in red to guide the eye.

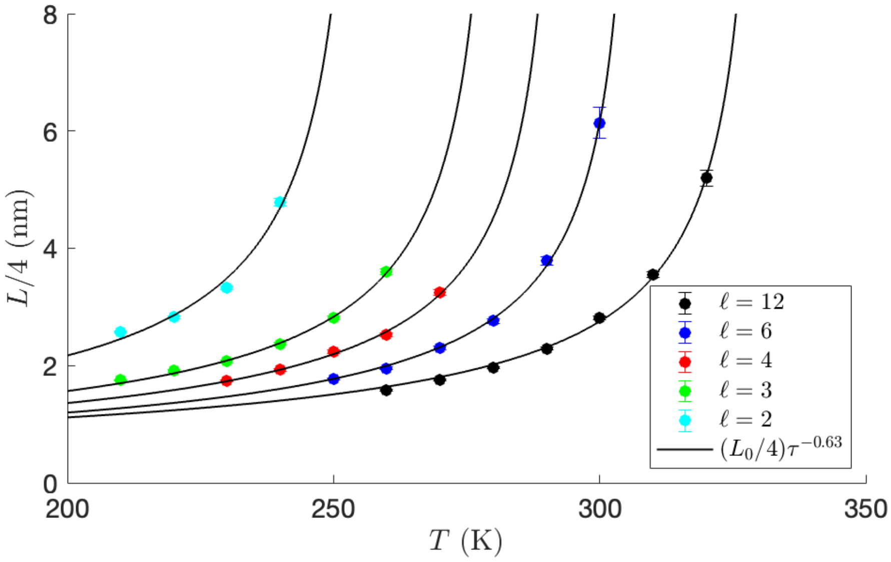

Fig. S7. Power-law behavior of interface width. Interface widths calculated from density profiles of five independent simulation runs performed as in Fig. 2 of the main text are shown as data points. For each block length ℓ, the fit to the expected power law with respect to reduced temperature ($L \sim \tau^{-0.63}$) is shown in black. The error bars indicate the standard error of the mean over simulation runs.

SI References

1. L. P. Kadanoff, *et al.*, Static Phenomena Near Critical Points: Theory and Experiment. *Rev. Mod. Phys.* **39**, 395-431 (1967).
2. G. Galliero, Surface tension of short flexible Lennard-Jones chains: Corresponding states behavior. *J. Chem. Phys.* **133**, 074705 (2010).
